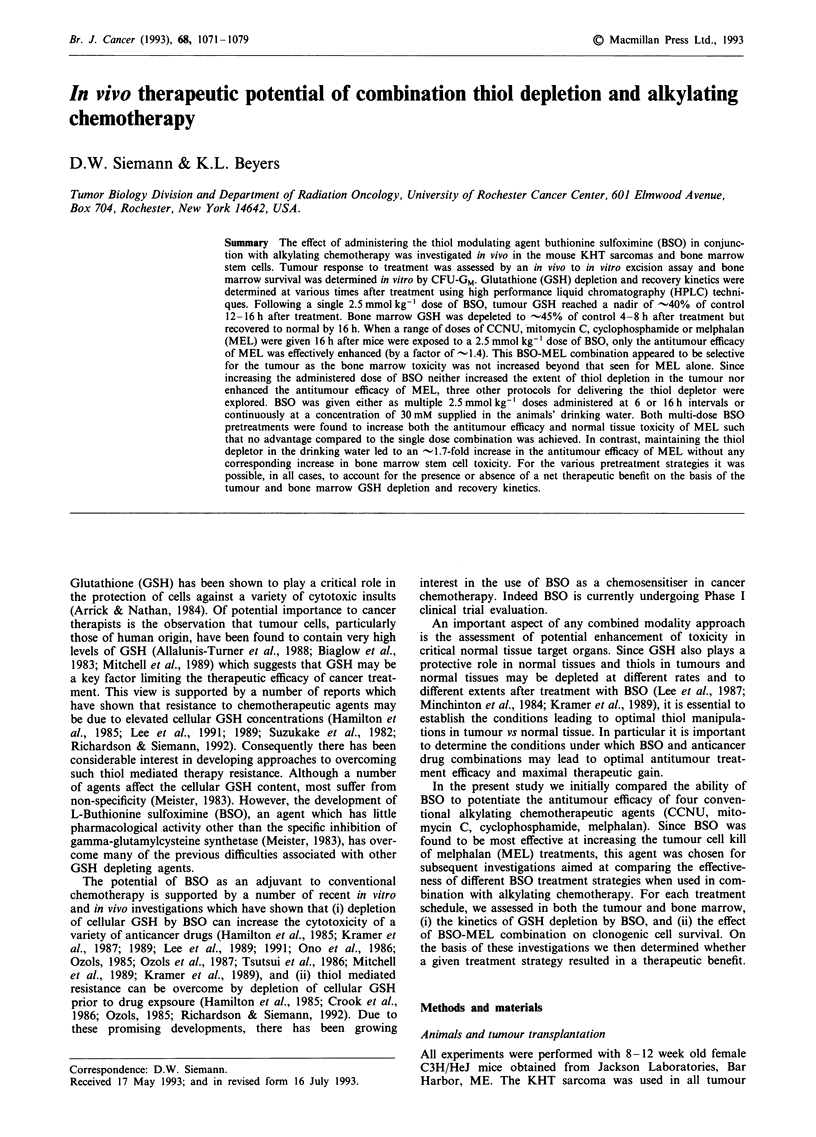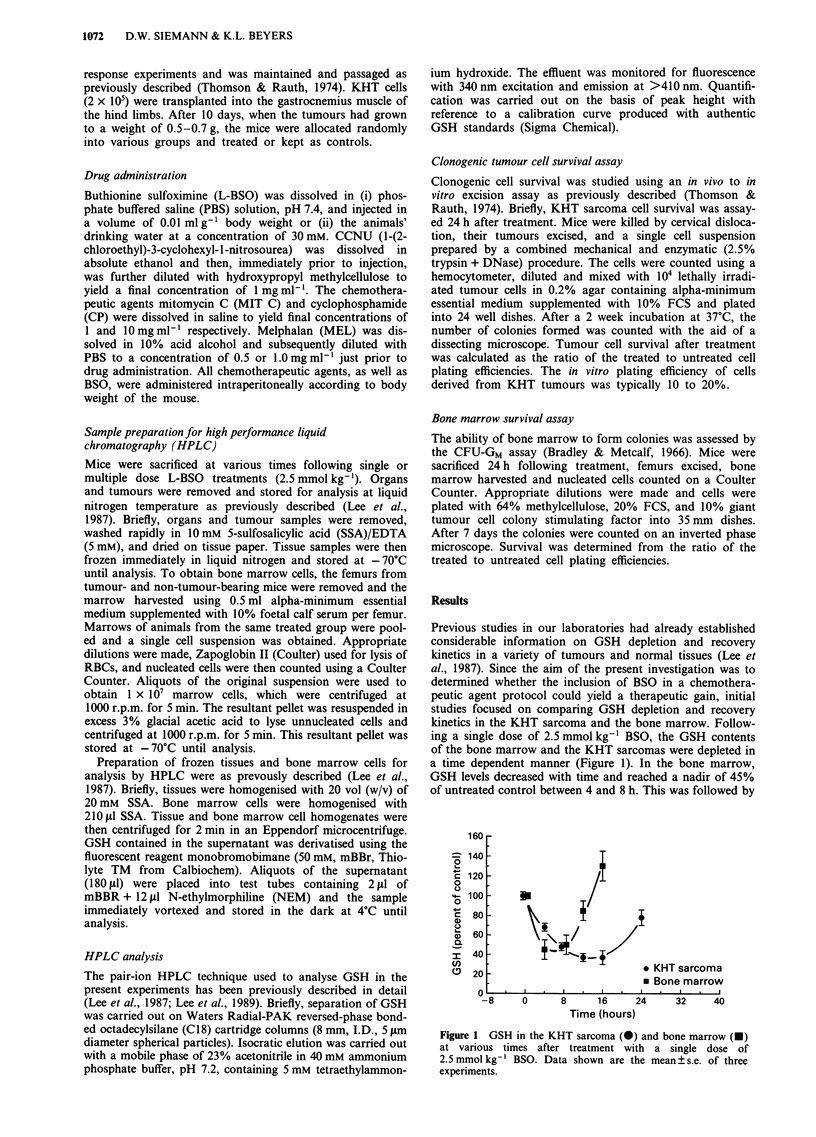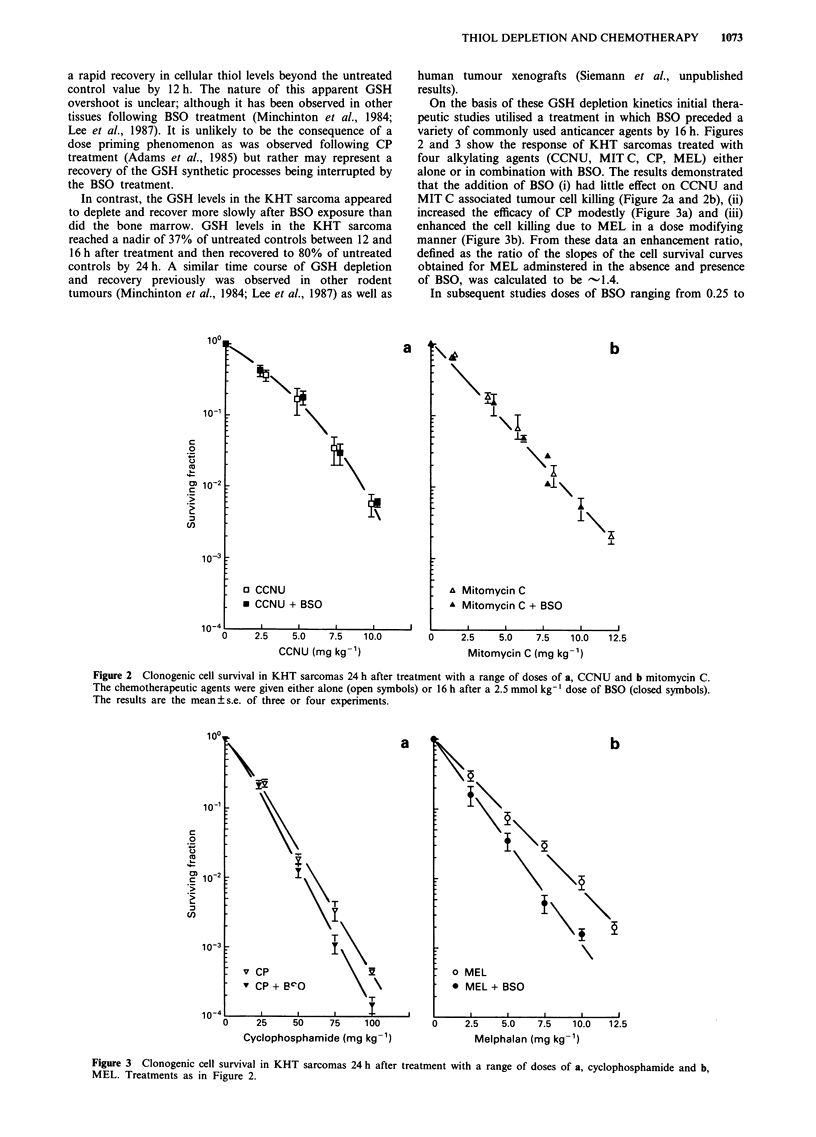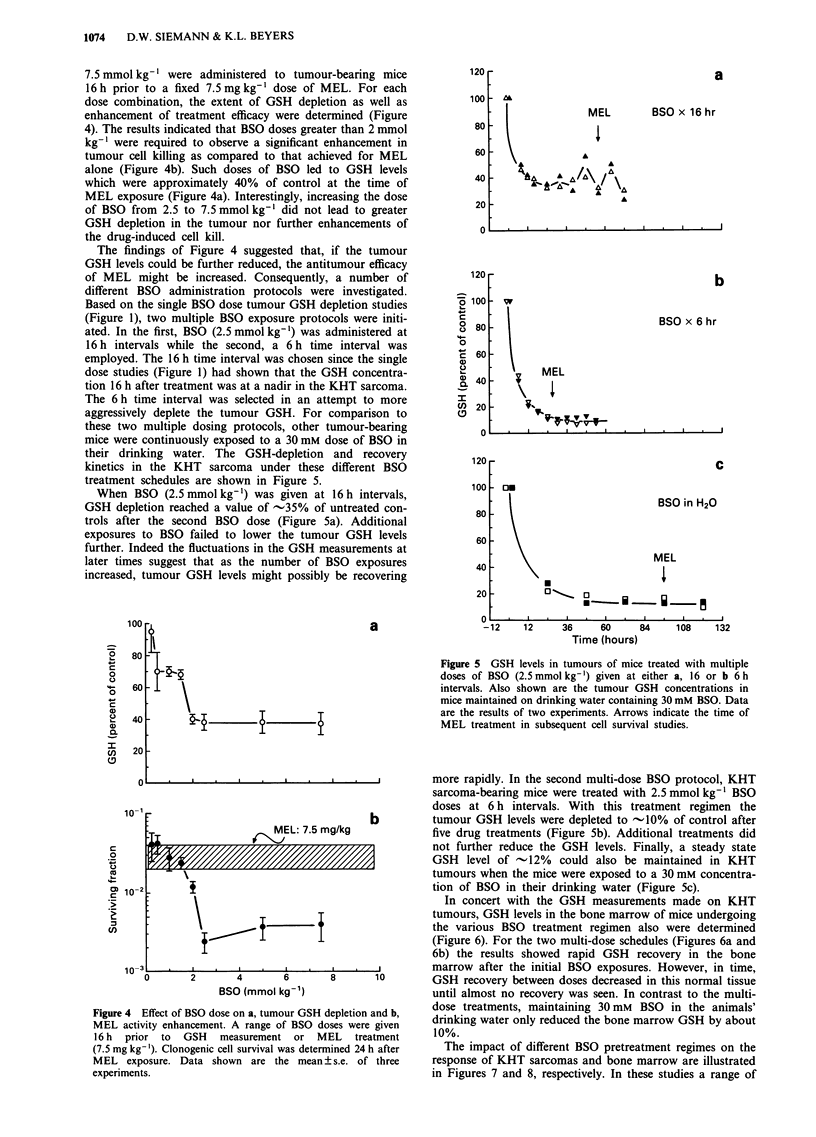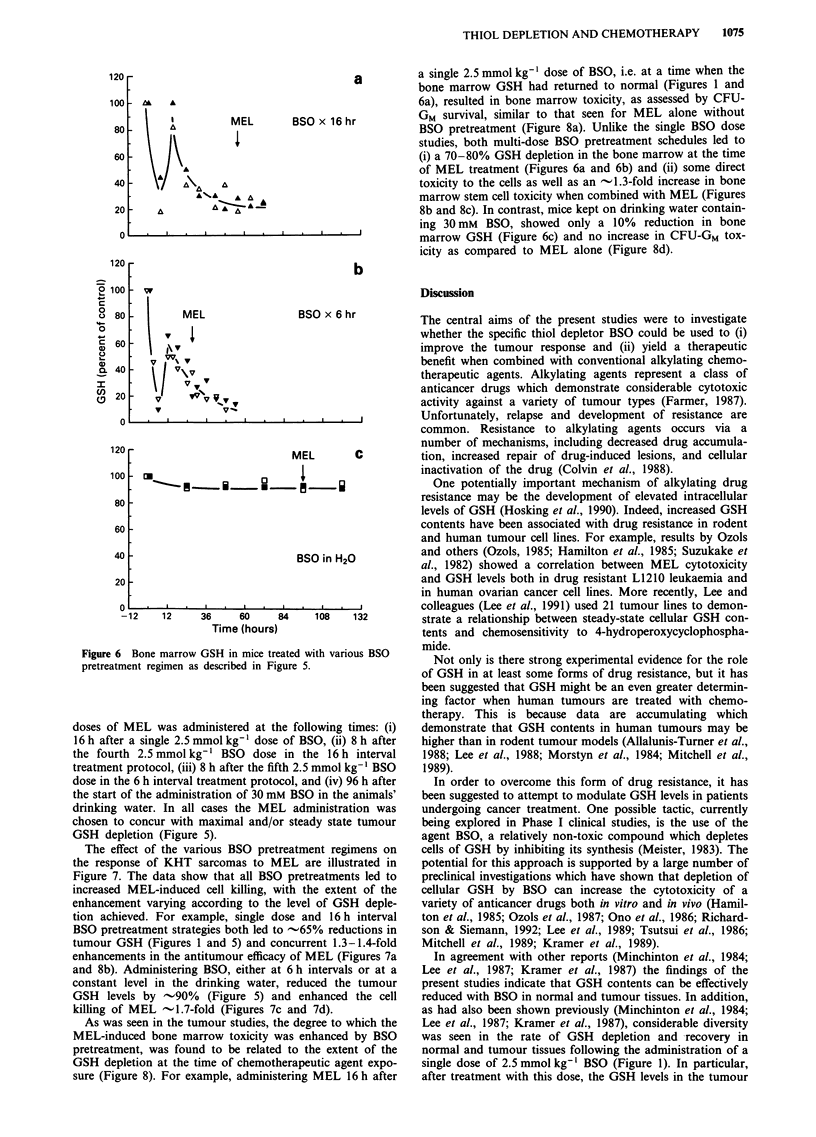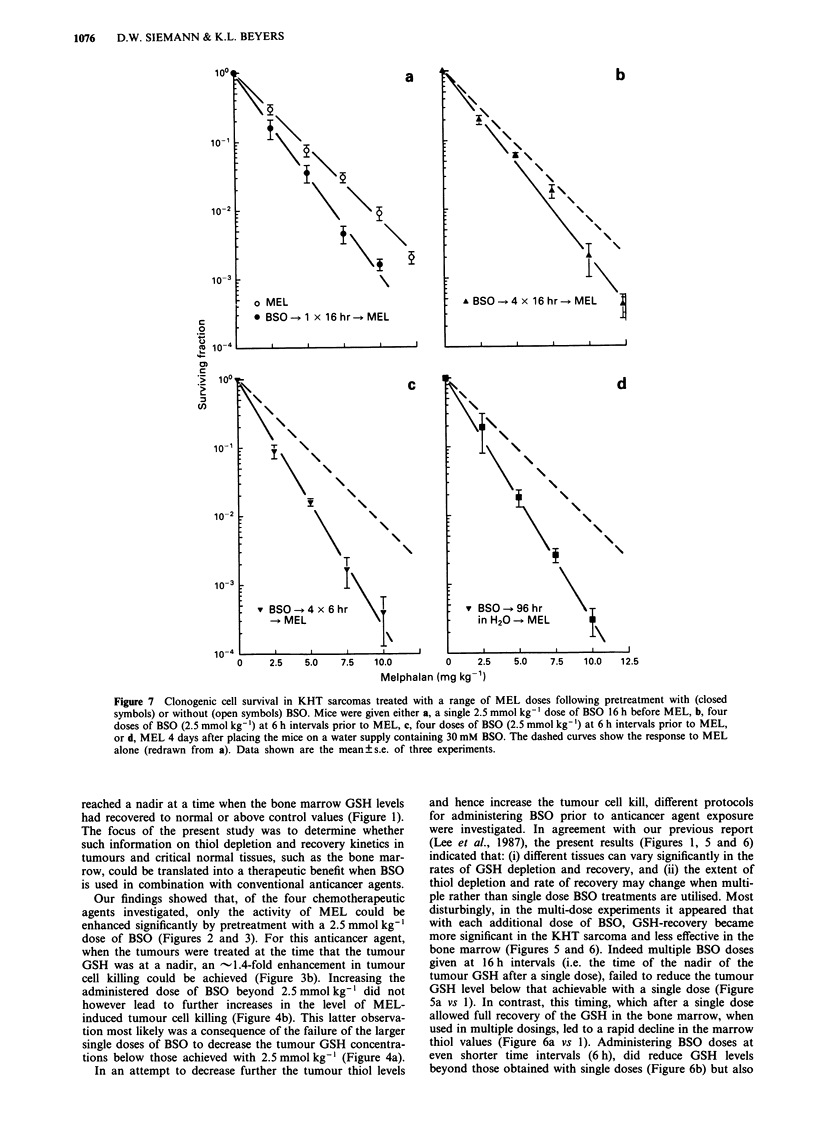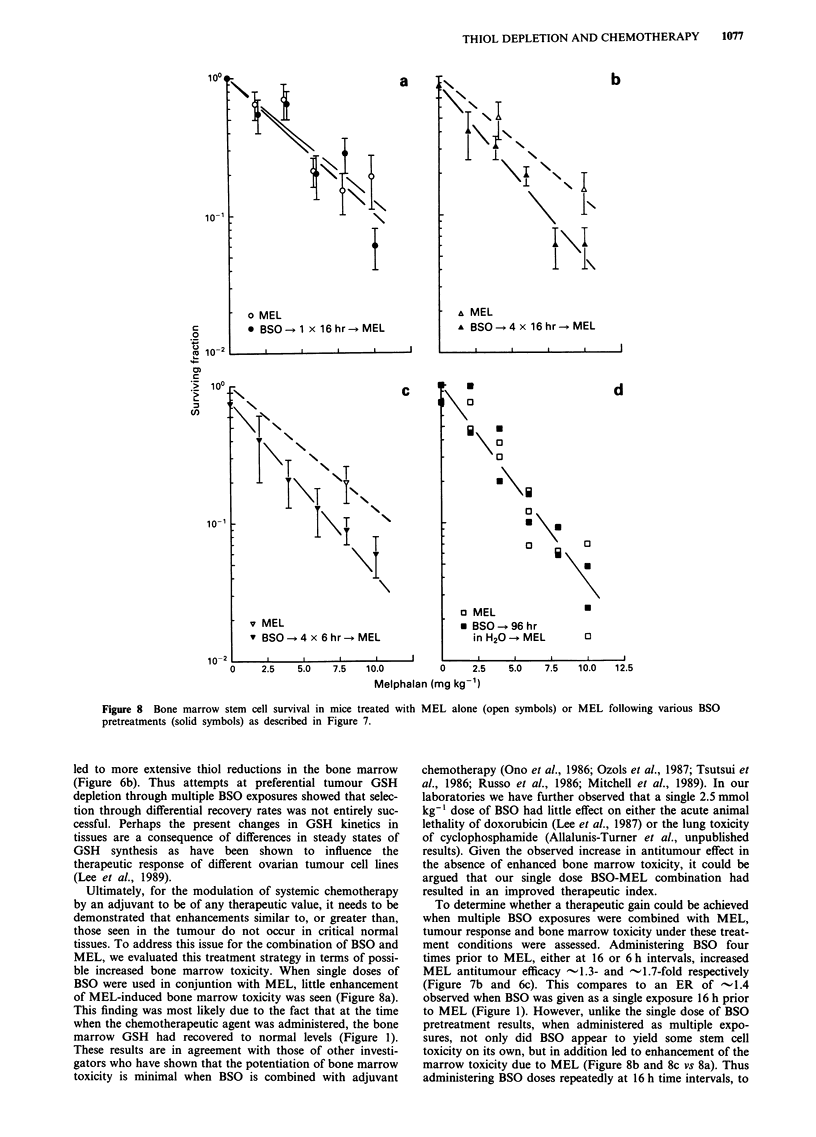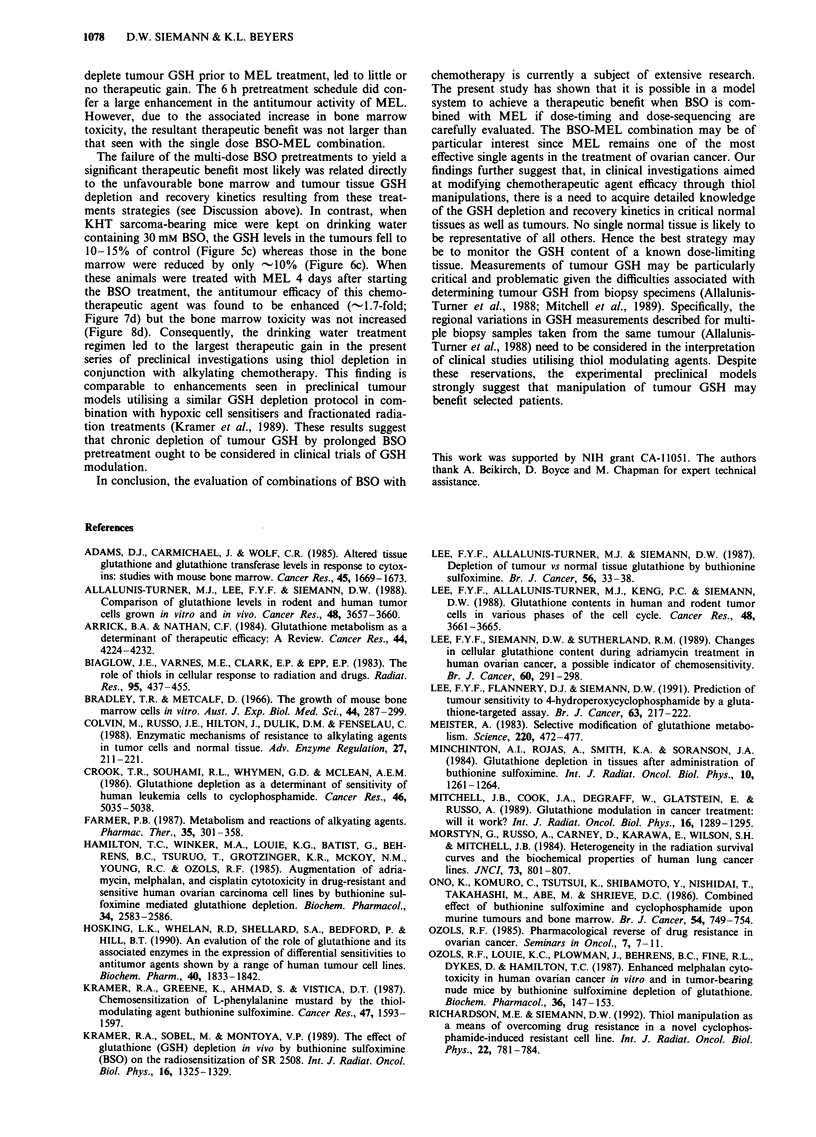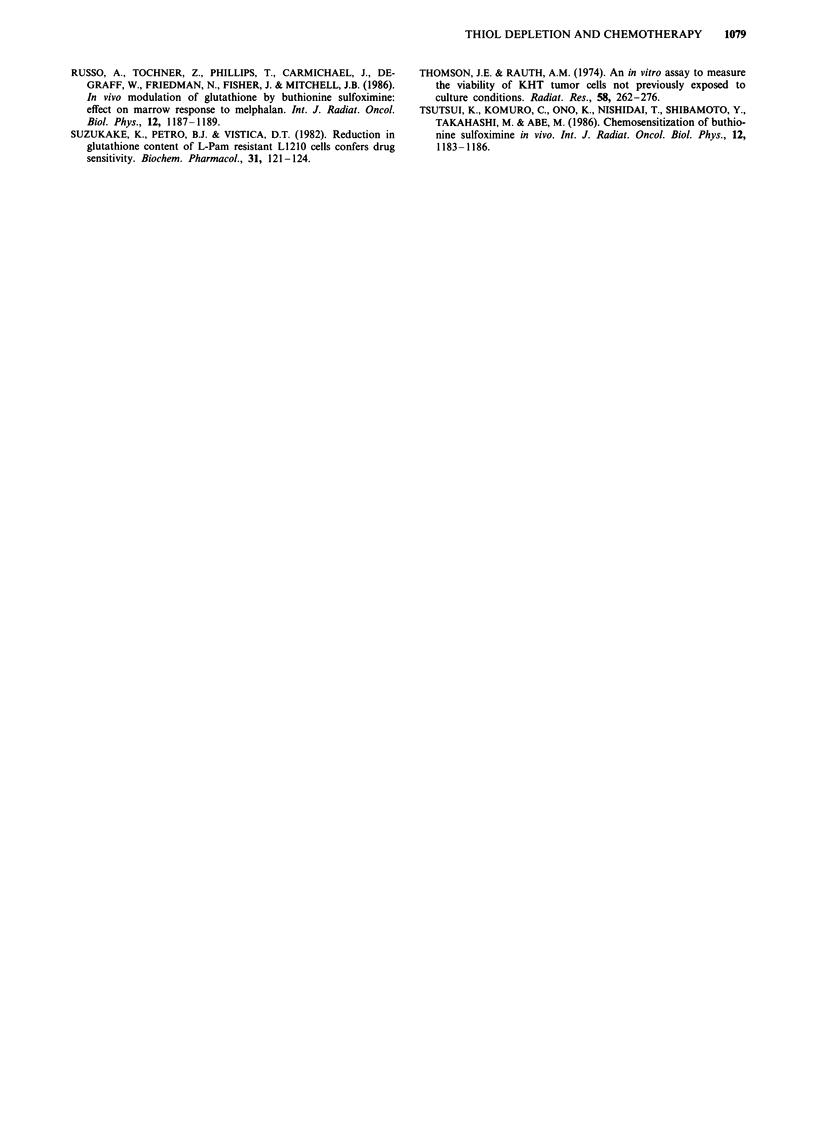# In vivo therapeutic potential of combination thiol depletion and alkylating chemotherapy.

**DOI:** 10.1038/bjc.1993.484

**Published:** 1993-12

**Authors:** D. W. Siemann, K. L. Beyers

**Affiliations:** Tumor Biology Division, University of Rochester Cancer Center, New York 14642.

## Abstract

The effect of administering the thiol modulating agent buthionine sulfoximine (BSO) in conjunction with alkylating chemotherapy was investigated in vivo in the mouse KHT sarcomas and bone marrow stem cells. Tumour response to treatment was assessed by an in vivo to in vitro excision assay and bone marrow survival was determined in vitro by CFU-GM. Glutathione (GSH) depletion and recovery kinetics were determined at various times after treatment using high performance liquid chromatography (HPLC) techniques. Following a single 2.5 mmol kg-1 dose of BSO, tumour GSH reached a nadir of approximately 40% of control 12-16 h after treatment. Bone marrow GSH was depleted to approximately 45% of control 4-8 h after treatment but recovered to normal by 16 h. When a range of doses of CCNU, mitomycin C, cyclophosphamide or melphalan (MEL) were given 16 h after mice were exposed to a 2.5 mmol kg-1 dose of BSO, only the antitumour efficacy of MEL was effectively enhanced (by a factor of approximately 1.4). This BSO-MEL combination appeared to be selective for the tumour as the bone marrow toxicity was not increased beyond that seen for MEL alone. Since increasing the administered dose of BSO neither increased the extent of thiol depletion in the tumour nor enhanced the antitumour efficacy of MEL, three other protocols for delivering the thiol depletor were explored. BSO was given either as multiple 2.5 mmol kg-1 doses administered at 6 or 16 h intervals or continuously at a concentration of 30 mM supplied in the animals' drinking water. Both multi-dose BSO pretreatments were found to increase both the antitumour efficacy and normal tissue toxicity of MEL such that no advantage compared to the single dose combination was achieved. In contrast, maintaining the thiol depletor in the drinking water led to an approximately 1.7-fold increase in the antitumour efficacy of MEL without any corresponding increase in bone marrow stem cell toxicity. For the various pretreatment strategies it was possible, in all cases, to account for the presence or absence of a net therapeutic benefit on the basis of the tumour and bone marrow GSH depletion and recovery kinetics.


Br. J. Cancer (1993), 68, 1071–1079 © Macmillan Press Ltd., 1993

### *In vivo* therapeutic potential of combination thiol depletion and alkylating chemotherapy

D.W. Siemann & K.L. Beyers

*Tumor Biology Division and Department of Radiation Oncology, University of Rochester Cancer Center, 601 Elmwood Avenue, Box 704, Rochester, New York 14642, USA.*

**Summary** The effect of administering the thiol modulating agent buthionine sulfoximine (BSO) in conjunction with alkylating chemotherapy was investigated *in vivo* in the mouse KHT sarcomas and bone marrow stem cells. Tumour response to treatment was assessed by an *in vivo* to *in vitro* excision assay and bone marrow survival was determined *in vitro* by CFU-$G_M$. Glutathione (GSH) depletion and recovery kinetics were determined at various times after treatment using high performance liquid chromatography (HPLC) techniques. Following a single 2.5 mmol kg$^{-1}$ dose of BSO, tumour GSH reached a nadir of ~40% of control 12–16 h after treatment. Bone marrow GSH was depeleted to ~45% of control 4–8 h after treatment but recovered to normal by 16 h. When a range of doses of CCNU, mitomycin C, cyclophosphamide or melphalan (MEL) were given 16 h after mice were exposed to a 2.5 mmol kg$^{-1}$ dose of BSO, only the antitumour efficacy of MEL was effectively enhanced (by a factor of ~1.4). This BSO-MEL combination appeared to be selective for the tumour as the bone marrow toxicity was not increased beyond that seen for MEL alone. Since increasing the administered dose of BSO neither increased the extent of thiol depletion in the tumour nor enhanced the antitumour efficacy of MEL, three other protocols for delivering the thiol depletor were explored. BSO was given either as multiple 2.5 mmol kg$^{-1}$ doses administered at 6 or 16 h intervals or continuously at a concentration of 30 mM supplied in the animals' drinking water. Both multi-dose BSO pretreatments were found to increase both the antitumour efficacy and normal tissue toxicity of MEL such that no advantage compared to the single dose combination was achieved. In contrast, maintaining the thiol depletor in the drinking water led to an ~1.7-fold increase in the antitumour efficacy of MEL without any corresponding increase in bone marrow stem cell toxicity. For the various pretreatment strategies it was possible, in all cases, to account for the presence or absence of a net therapeutic benefit on the basis of the tumour and bone marrow GSH depletion and recovery kinetics.

Glutathione (GSH) has been shown to play a critical role in the protection of cells against a variety of cytotoxic insults (Arrick & Nathan, 1984). Of potential importance to cancer therapists is the observation that tumour cells, particularly those of human origin, have been found to contain very high levels of GSH (Allalunis-Turner *et al.*, 1988; Biaglow *et al.*, 1983; Mitchell *et al.*, 1989) which suggests that GSH may be a key factor limiting the therapeutic efficacy of cancer treatment. This view is supported by a number of reports which have shown that resistance to chemotherapeutic agents may be due to elevated cellular GSH concentrations (Hamilton *et al.*, 1985; Lee *et al.*, 1991; 1989; Suzukake *et al.*, 1982; Richardson & Siemann, 1992). Consequently there has been considerable interest in developing approaches to overcoming such thiol mediated therapy resistance. Although a number of agents affect the cellular GSH content, most suffer from non-specificity (Meister, 1983). However, the development of L-Buthionine sulfoximine (BSO), an agent which has little pharmacological activity other than the specific inhibition of gamma-glutamylcysteine synthetase (Meister, 1983), has overcome many of the previous difficulties associated with other GSH depleting agents.

The potential of BSO as an adjuvant to conventional chemotherapy is supported by a number of recent *in vitro* and *in vivo* investigations which have shown that (i) depletion of cellular GSH by BSO can increase the cytotoxicity of a variety of anticancer drugs (Hamilton *et al.*, 1985; Kramer *et al.*, 1987; 1989; Lee *et al.*, 1989; 1991; Ono *et al.*, 1986; Ozols, 1985; Ozols *et al.*, 1987; Tsutsui *et al.*, 1986; Mitchell *et al.*, 1989; Kramer *et al.*, 1989), and (ii) thiol mediated resistance can be overcome by depletion of cellular GSH prior to drug expsoure (Hamilton *et al.*, 1985; Crook *et al.*, 1986; Ozols, 1985; Richardson & Siemann, 1992). Due to these promising developments, there has been growing interest in the use of BSO as a chemosensitiser in cancer chemotherapy. Indeed BSO is currently undergoing Phase I clinical trial evaluation.

An important aspect of any combined modality approach is the assessment of potential enhancement of toxicity in critical normal tissue target organs. Since GSH also plays a protective role in normal tissues and thiols in tumours and normal tissues may be depleted at different rates and to different extents after treatment with BSO (Lee *et al.*, 1987; Minchinton *et al.*, 1984; Kramer *et al.*, 1989), it is essential to establish the conditions leading to optimal thiol manipulations in tumour *vs* normal tissue. In particular it is important to determine the conditions under which BSO and anticancer drug combinations may lead to optimal antitumour treatment efficacy and maximal therapeutic gain.

In the present study we initially compared the ability of BSO to potentiate the antitumour efficacy of four conventional alkylating chemotherapeutic agents (CCNU, mitomycin C, cyclophosphamide, melphalan). Since BSO was found to be most effective at increasing the tumour cell kill of melphalan (MEL) treatments, this agent was chosen for subsequent investigations aimed at comparing the effectiveness of different BSO treatment strategies when used in combination with alkylating chemotherapy. For each treatment schedule, we assessed in both the tumour and bone marrow, (i) the kinetics of GSH depletion by BSO, and (ii) the effect of BSO-MEL combination on clonogenic cell survival. On the basis of these investigations we then determined whether a given treatment strategy resulted in a therapeutic benefit.

### Methods and materials

### *Animals and tumour transplantation*

All experiments were performed with 8–12 week old female C3H/HeJ mice obtained from Jackson Laboratories, Bar Harbor, ME. The KHT sarcoma was used in all tumour

Correspondence: D.W. Siemann.
Received 17 May 1993; and in revised form 16 July 1993.

response experiments and was maintained and passaged as previously described (Thomson & Rauth, 1974). KHT cells ($2 \times 10^5$) were transplanted into the gastrocnemius muscle of the hind limbs. After 10 days, when the tumours had grown to a weight of 0.5–0.7 g, the mice were allocated randomly into various groups and treated or kept as controls.

*Drug administration*

Buthionine sulfoximine (L-BSO) was dissolved in (i) phosphate buffered saline (PBS) solution, pH 7.4, and injected in a volume of 0.01 ml $g^{-1}$ body weight or (ii) the animals' drinking water at a concentration of 30 mM. CCNU (1-(2-chloroethyl)-3-cyclohexyl-1-nitrosourea) was dissolved in absolute ethanol and then, immediately prior to injection, was further diluted with hydroxypropyl methylcellulose to yield a final concentration of 1 mg $ml^{-1}$. The chemotherapeutic agents mitomycin C (MIT C) and cyclophosphamide (CP) were dissolved in saline to yield final concentrations of 1 and 10 mg $ml^{-1}$ respectively. Melphalan (MEL) was dissolved in 10% acid alcohol and subsequently diluted with PBS to a concentration of 0.5 or 1.0 mg $ml^{-1}$ just prior to drug administration. All chemotherapeutic agents, as well as BSO, were administered intraperitoneally according to body weight of the mouse.

*Sample preparation for high performance liquid chromatography (HPLC)*

Mice were sacrificed at various times following single or multiple dose L-BSO treatments (2.5 mmol $kg^{-1}$). Organs and tumours were removed and stored for analysis at liquid nitrogen temperature as previously described (Lee *et al.*, 1987). Briefly, organs and tumour samples were removed, washed rapidly in 10 mM 5-sulfosalicylic acid (SSA)/EDTA (5 mM), and dried on tissue paper. Tissue samples were then frozen immediately in liquid nitrogen and stored at −70°C until analysis. To obtain bone marrow cells, the femurs from tumour- and non-tumour-bearing mice were removed and the marrow harvested using 0.5 ml alpha-minimum essential medium supplemented with 10% foetal calf serum per femur. Marrows of animals from the same treated group were pooled and a single cell suspension was obtained. Appropriate dilutions were made, Zapoglobin II (Coulter) used for lysis of RBCs, and nucleated cells were then counted using a Coulter Counter. Aliquots of the original suspension were used to obtain $1 \times 10^7$ marrow cells, which were centrifuged at 1000 r.p.m. for 5 min. The resultant pellet was resuspended in excess 3% glacial acetic acid to lyse unnucleated cells and centrifuged at 1000 r.p.m. for 5 min. This resultant pellet was stored at −70°C until analysis.

Preparation of frozen tissues and bone marrow cells for analysis by HPLC were as prevously described (Lee *et al.*, 1987). Briefly, tissues were homogenised with 20 vol (w/v) of 20 mM SSA. Bone marrow cells were homogenised with 210 µl SSA. Tissue and bone marrow cell homogenates were then centrifuged for 2 min in an Eppendorf microcentrifuge. GSH contained in the supernatant was derivatised using the fluorescent reagent monobromobimane (50 mM, mBBr, Thiolyte TM from Calbiochem). Aliquots of the supernatant (180 µl) were placed into test tubes containing 2 µl of mBBR + 12 µl N-ethylmorphiline (NEM) and the sample immediately vortexed and stored in the dark at 4°C until analysis.

*HPLC analysis*

The pair-ion HPLC technique used to analyse GSH in the present experiments has been previously described in detail (Lee *et al.*, 1987; Lee *et al.*, 1989). Briefly, separation of GSH was carried out on Waters Radial-PAK reversed-phase bonded octadecylsilane (C18) cartridge columns (8 mm, I.D., 5 µm diameter spherical particles). Isocratic elution was carried out with a mobile phase of 23% acetonitrile in 40 mM ammonium phosphate buffer, pH 7.2, containing 5 mM tetraethylammonium hydroxide. The effluent was monitored for fluorescence with 340 nm excitation and emission at >410 nm. Quantification was carried out on the basis of peak height with reference to a calibration curve produced with authentic GSH standards (Sigma Chemical).

*Clonogenic tumour cell survival assay*

Clonogenic cell survival was studied using an *in vivo* to *in vitro* excision assay as previously described (Thomson & Rauth, 1974). Briefly, KHT sarcoma cell survival was assayed 24 h after treatment. Mice were killed by cervical dislocation, their tumours excised, and a single cell suspension prepared by a combined mechanical and enzymatic (2.5% trypsin + DNase) procedure. The cells were counted using a hemocytometer, diluted and mixed with $10^4$ lethally irradiated tumour cells in 0.2% agar containing alpha-minimum essential medium supplemented with 10% FCS and plated into 24 well dishes. After a 2 week incubation at 37°C, the number of colonies formed was counted with the aid of a dissecting microscope. Tumour cell survival after treatment was calculated as the ratio of the treated to untreated cell plating efficiencies. The *in vitro* plating efficiency of cells derived from KHT tumours was typically 10 to 20%.

*Bone marrow survival assay*

The ability of bone marrow to form colonies was assessed by the CFU-$G_M$ assay (Bradley & Metcalf, 1966). Mice were sacrificed 24 h following treatment, femurs excised, bone marrow harvested and nucleated cells counted on a Coulter Counter. Appropriate dilutions were made and cells were plated with 64% methylcellulose, 20% FCS, and 10% giant tumour cell colony stimulating factor into 35 mm dishes. After 7 days the colonies were counted on an inverted phase microscope. Survival was determined from the ratio of the treated to untreated cell plating efficiencies.

### Results

Previous studies in our laboratories had already established considerable information on GSH depletion and recovery kinetics in a variety of tumours and normal tissues (Lee *et al.*, 1987). Since the aim of the present investigation was to determined whether the inclusion of BSO in a chemotherapeutic agent protocol could yield a therapeutic gain, initial studies focused on comparing GSH depletion and recovery kinetics in the KHT sarcoma and the bone marrow. Following a single dose of 2.5 mmol $kg^{-1}$ BSO, the GSH contents of the bone marrow and the KHT sarcomas were depleted in a time dependent manner (Figure 1). In the bone marrow, GSH levels decreased with time and reached a nadir of 45% of untreated control between 4 and 8 h. This was followed by

**Figure 1** GSH in the KHT sarcoma (●) and bone marrow (■) at various times after treatment with a single dose of 2.5 mmol $kg^{-1}$ BSO. Data shown are the mean ± s.e. of three experiments.

a rapid recovery in cellular thiol levels beyond the untreated control value by 12 h. The nature of this apparent GSH overshoot is unclear; although it has been observed in other tissues following BSO treatment (Minchinton *et al.*, 1984; Lee *et al.*, 1987). It is unlikely to be the consequence of a dose priming phenomenon as was observed following CP treatment (Adams *et al.*, 1985) but rather may represent a recovery of the GSH synthetic processes being interrupted by the BSO treatment.

In contrast, the GSH levels in the KHT sarcoma appeared to deplete and recover more slowly after BSO exposure than did the bone marrow. GSH levels in the KHT sarcoma reached a nadir of 37% of untreated controls between 12 and 16 h after treatment and then recovered to 80% of untreated controls by 24 h. A similar time course of GSH depletion and recovery previously was observed in other rodent tumours (Minchinton *et al.*, 1984; Lee *et al.*, 1987) as well as human tumour xenografts (Siemann *et al.*, unpublished results).

On the basis of these GSH depletion kinetics initial therapeutic studies utilised a treatment in which BSO preceded a variety of commonly used anticancer agents by 16 h. Figures 2 and 3 show the response of KHT sarcomas treated with four alkylating agents (CCNU, MIT C, CP, MEL) either alone or in combination with BSO. The results demonstrated that the addition of BSO (i) had little effect on CCNU and MIT C associated tumour cell killing (Figure 2a and 2b), (ii) increased the efficacy of CP modestly (Figure 3a) and (iii) enhanced the cell killing due to MEL in a dose modifying manner (Figure 3b). From these data an enhancement ratio, defined as the ratio of the slopes of the cell survival curves obtained for MEL adminstered in the absence and presence of BSO, was calculated to be ~1.4.

In subsequent studies doses of BSO ranging from 0.25 to

**Figure 2** Clonogenic cell survival in KHT sarcomas 24 h after treatment with a range of doses of **a**, CCNU and **b** mitomycin C. The chemotherapeutic agents were given either alone (open symbols) or 16 h after a 2.5 mmol kg$^{-1}$ dose of BSO (closed symbols). The results are the mean ± s.e. of three or four experiments.

**Figure 3** Clonogenic cell survival in KHT sarcomas 24 h after treatment with a range of doses of **a**, cyclophosphamide and **b**, MEL. Treatments as in Figure 2.

7.5 mmol kg$^{-1}$ were administered to tumour-bearing mice 16 h prior to a fixed 7.5 mg kg$^{-1}$ dose of MEL. For each dose combination, the extent of GSH depletion as well as enhancement of treatment efficacy were determined (Figure 4). The results indicated that BSO doses greater than 2 mmol kg$^{-1}$ were required to observe a significant enhancement in tumour cell killing as compared to that achieved for MEL alone (Figure 4b). Such doses of BSO led to GSH levels which were approximately 40% of control at the time of MEL exposure (Figure 4a). Interestingly, increasing the dose of BSO from 2.5 to 7.5 mmol kg$^{-1}$ did not lead to greater GSH depletion in the tumour nor further enhancements of the drug-induced cell kill.

The findings of Figure 4 suggested that, if the tumour GSH levels could be further reduced, the antitumour efficacy of MEL might be increased. Consequently, a number of different BSO administration protocols were investigated. Based on the single BSO dose tumour GSH depletion studies (Figure 1), two multiple BSO exposure protocols were initiated. In the first, BSO (2.5 mmol kg$^{-1}$) was administered at 16 h intervals while the second, a 6 h time interval was employed. The 16 h time interval was chosen since the single dose studies (Figure 1) had shown that the GSH concentration 16 h after treatment was at a nadir in the KHT sarcoma. The 6 h time interval was selected in an attempt to more aggressively deplete the tumour GSH. For comparison to these two multiple dosing protocols, other tumour-bearing mice were continuously exposed to a 30 mM dose of BSO in their drinking water. The GSH-depletion and recovery kinetics in the KHT sarcoma under these different BSO treatment schedules are shown in Figure 5.

When BSO (2.5 mmol kg$^{-1}$) was given at 16 h intervals, GSH depletion reached a value of ~35% of untreated controls after the second BSO dose (Figure 5a). Additional exposures to BSO failed to lower the tumour GSH levels further. Indeed the fluctuations in the GSH measurements at later times suggest that as the number of BSO exposures increased, tumour GSH levels might possibly be recovering

**Figure 4** Effect of BSO dose on **a**, tumour GSH depletion and **b**, MEL activity enhancement. A range of BSO doses were given 16 h prior to GSH measurement or MEL treatment (7.5 mg kg$^{-1}$). Clonogenic cell survival was determined 24 h after MEL exposure. Data shown are the mean ± s.e. of three experiments.

**Figure 5** GSH levels in tumours of mice treated with multiple doses of BSO (2.5 mmol kg$^{-1}$) given at either **a**, 16 or **b** 6 h intervals. Also shown are the tumour GSH concentrations in mice maintained on drinking water containing 30 mM BSO. Data are the results of two experiments. Arrows indicate the time of MEL treatment in subsequent cell survival studies.

more rapidly. In the second multi-dose BSO protocol, KHT sarcoma-bearing mice were treated with 2.5 mmol kg$^{-1}$ BSO doses at 6 h intervals. With this treatment regimen the tumour GSH levels were depleted to ~10% of control after five drug treatments (Figure 5b). Additional treatments did not further reduce the GSH levels. Finally, a steady state GSH level of ~12% could also be maintained in KHT tumours when the mice were exposed to a 30 mM concentration of BSO in their drinking water (Figure 5c).

In concert with the GSH measurements made on KHT tumours, GSH levels in the bone marrow of mice undergoing the various BSO treatment regimen also were determined (Figure 6). For the two multi-dose schedules (Figures 6a and 6b) the results showed rapid GSH recovery in the bone marrow after the initial BSO exposures. However, in time, GSH recovery between doses decreased in this normal tissue until almost no recovery was seen. In contrast to the multi-dose treatments, maintaining 30 mM BSO in the animals' drinking water only reduced the bone marrow GSH by about 10%.

The impact of different BSO pretreatment regimes on the response of KHT sarcomas and bone marrow are illustrated in Figures 7 and 8, respectively. In these studies a range of

**Figure 6** Bone marrow GSH in mice treated with various BSO pretreatment regimen as described in Figure 5.

doses of MEL was administered at the following times: (i) 16 h after a single 2.5 mmol $kg^{-1}$ dose of BSO, (ii) 8 h after the fourth 2.5 mmol $kg^{-1}$ BSO dose in the 16 h interval treatment protocol, (iii) 8 h after the fifth 2.5 mmol $kg^{-1}$ BSO dose in the 6 h interval treatment protocol, and (iv) 96 h after the start of the administration of 30 mM BSO in the animals' drinking water. In all cases the MEL administration was chosen to concur with maximal and/or steady state tumour GSH depletion (Figure 5).

The effect of the various BSO pretreatment regimens on the response of KHT sarcomas to MEL are illustrated in Figure 7. The data show that all BSO pretreatments led to increased MEL-induced cell killing, with the extent of the enhancement varying according to the level of GSH depletion achieved. For example, single dose and 16 h interval BSO pretreatment strategies both led to ~65% reductions in tumour GSH (Figures 1 and 5) and concurrent 1.3-1.4-fold enhancements in the antitumour efficacy of MEL (Figures 7a and 8b). Administering BSO, either at 6 h intervals or at a constant level in the drinking water, reduced the tumour GSH levels by ~90% (Figure 5) and enhanced the cell killing of MEL ~1.7-fold (Figures 7c and 7d).

As was seen in the tumour studies, the degree to which the MEL-induced bone marrow toxicity was enhanced by BSO pretreatment, was found to be related to the extent of the GSH depletion at the time of chemotherapeutic agent exposure (Figure 8). For example, administering MEL 16 h after a single 2.5 mmol $kg^{-1}$ dose of BSO, i.e. at a time when the bone marrow GSH had returned to normal (Figures 1 and 6a), resulted in bone marrow toxicity, as assessed by CFU-$G_M$ survival, similar to that seen for MEL alone without BSO pretreatment (Figure 8a). Unlike the single BSO dose studies, both multi-dose BSO pretreatment schedules led to (i) a 70-80% GSH depletion in the bone marrow at the time of MEL treatment (Figures 6a and 6b) and (ii) some direct toxicity to the cells as well as an ~1.3-fold increase in bone marrow stem cell toxicity when combined with MEL (Figures 8b and 8c). In contrast, mice kept on drinking water containing 30 mM BSO, showed only a 10% reduction in bone marrow GSH (Figure 6c) and no increase in CFU-$G_M$ toxicity as compared to MEL alone (Figure 8d).

### Discussion

The central aims of the present studies were to investigate whether the specific thiol depletor BSO could be used to (i) improve the tumour response and (ii) yield a therapeutic benefit when combined with conventional alkylating chemotherapeutic agents. Alkylating agents represent a class of anticancer drugs which demonstrate considerable cytotoxic activity against a variety of tumour types (Farmer, 1987). Unfortunately, relapse and development of resistance are common. Resistance to alkylating agents occurs via a number of mechanisms, including decreased drug accumulation, increased repair of drug-induced lesions, and cellular inactivation of the drug (Colvin *et al.*, 1988).

One potentially important mechanism of alkylating drug resistance may be the development of elevated intracellular levels of GSH (Hosking *et al.*, 1990). Indeed, increased GSH contents have been associated with drug resistance in rodent and human tumour cell lines. For example, results by Ozols and others (Ozols, 1985; Hamilton *et al.*, 1985; Suzukake *et al.*, 1982) showed a correlation between MEL cytotoxicity and GSH levels both in drug resistant L1210 leukaemia and in human ovarian cancer cell lines. More recently, Lee and colleagues (Lee *et al.*, 1991) used 21 tumour lines to demonstrate a relationship between steady-state cellular GSH contents and chemosensitivity to 4-hydroperoxycyclophosphamide.

Not only is there strong experimental evidence for the role of GSH in at least some forms of drug resistance, but it has been suggested that GSH might be an even greater determining factor when human tumours are treated with chemotherapy. This is because data are accumulating which demonstrate that GSH contents in human tumours may be higher than in rodent tumour models (Allalunis-Turner *et al.*, 1988; Lee *et al.*, 1988; Morstyn *et al.*, 1984; Mitchell *et al.*, 1989).

In order to overcome this form of drug resistance, it has been suggested to attempt to modulate GSH levels in patients undergoing cancer treatment. One possible tactic, currently being explored in Phase I clinical studies, is the use of the agent BSO, a relatively non-toxic compound which depletes cells of GSH by inhibiting its synthesis (Meister, 1983). The potential for this approach is supported by a large number of preclinical investigations which have shown that depletion of cellular GSH by BSO can increase the cytotoxicity of a variety of anticancer drugs both *in vitro* and *in vivo* (Hamilton *et al.*, 1985; Ozols *et al.*, 1987; Ono *et al.*, 1986; Richardson & Siemann, 1992; Lee *et al.*, 1989; Tsutsui *et al.*, 1986; Mitchell *et al.*, 1989; Kramer *et al.*, 1989).

In agreement with other reports (Minchinton *et al.*, 1984; Lee *et al.*, 1987; Kramer *et al.*, 1987) the findings of the present studies indicate that GSH contents can be effectively reduced with BSO in normal and tumour tissues. In addition, as had also been shown previously (Minchinton *et al.*, 1984; Lee *et al.*, 1987; Kramer *et al.*, 1987), considerable diversity was seen in the rate of GSH depletion and recovery in normal and tumour tissues following the administration of a single dose of 2.5 mmol $kg^{-1}$ BSO (Figure 1). In particular, after treatment with this dose, the GSH levels in the tumour

**Figure 7** Clonogenic cell survival in KHT sarcomas treated with a range of MEL doses following pretreatment with (closed symbols) or without (open symbols) BSO. Mice were given either **a**, a single 2.5 mmol kg$^{-1}$ dose of BSO 16 h before MEL, **b**, four doses of BSO (2.5 mmol kg$^{-1}$) at 6 h intervals prior to MEL, **c**, four doses of BSO (2.5 mmol kg$^{-1}$) at 6 h intervals prior to MEL, or **d**, MEL 4 days after placing the mice on a water supply containing 30 mM BSO. The dashed curves show the response to MEL alone (redrawn from **a**). Data shown are the mean ± s.e. of three experiments.

reached a nadir at a time when the bone marrow GSH levels had recovered to normal or above control values (Figure 1). The focus of the present study was to determine whether such information on thiol depletion and recovery kinetics in tumours and critical normal tissues, such as the bone marrow, could be translated into a therapeutic benefit when BSO is used in combination with conventional anticancer agents.

Our findings showed that, of the four chemotherapeutic agents investigated, only the activity of MEL could be enhanced significantly by pretreatment with a 2.5 mmol kg$^{-1}$ dose of BSO (Figures 2 and 3). For this anticancer agent, when the tumours were treated at the time that the tumour GSH was at a nadir, an ~1.4-fold enhancement in tumour cell killing could be achieved (Figure 3b). Increasing the administered dose of BSO beyond 2.5 mmol kg$^{-1}$ did not however lead to further increases in the level of MEL-induced tumour cell killing (Figure 4b). This latter observation most likely was a consequence of the failure of the larger single doses of BSO to decrease the tumour GSH concentrations below those achieved with 2.5 mmol kg$^{-1}$ (Figure 4a).

In an attempt to decrease further the tumour thiol levels and hence increase the tumour cell kill, different protocols for administering BSO prior to anticancer agent exposure were investigated. In agreement with our previous report (Lee *et al.*, 1987), the present results (Figures 1, 5 and 6) indicated that: (i) different tissues can vary significantly in the rates of GSH depletion and recovery, and (ii) the extent of thiol depletion and rate of recovery may change when multiple rather than single dose BSO treatments are utilised. Most disturbingly, in the multi-dose experiments it appeared that with each additional dose of BSO, GSH-recovery became more significant in the KHT sarcoma and less effective in the bone marrow (Figures 5 and 6). Indeed multiple BSO doses given at 16 h intervals (i.e. the time of the nadir of the tumour GSH after a single dose), failed to reduce the tumour GSH level below that achievable with a single dose (Figure 5a *vs* 1). In contrast, this timing, which after a single dose allowed full recovery of the GSH in the bone marrow, when used in multiple dosings, led to a rapid decline in the marrow thiol values (Figure 6a *vs* 1). Administering BSO doses at even shorter time intervals (6 h), did reduce GSH levels beyond those obtained with single doses (Figure 6b) but also

**Figure 8** Bone marrow stem cell survival in mice treated with MEL alone (open symbols) or MEL following various BSO pretreatments (solid symbols) as described in Figure 7.

led to more extensive thiol reductions in the bone marrow (Figure 6b). Thus attempts at preferential tumour GSH depletion through multiple BSO exposures showed that selection through differential recovery rates was not entirely successful. Perhaps the present changes in GSH kinetics in tissues are a consequence of differences in steady states of GSH synthesis as have been shown to influence the therapeutic response of different ovarian tumour cell lines (Lee *et al.*, 1989).

Ultimately, for the modulation of systemic chemotherapy by an adjuvant to be of any therapeutic value, it needs to be demonstrated that enhancements similar to, or greater than, those seen in the tumour do not occur in critical normal tissues. To address this issue for the combination of BSO and MEL, we evaluated this treatment strategy in terms of possible increased bone marrow toxicity. When single doses of BSO were used in conjuntion with MEL, little enhancement of MEL-induced bone marrow toxicity was seen (Figure 8a). This finding was most likely due to the fact that at the time when the chemotherapeutic agent was administered, the bone marrow GSH had recovered to normal levels (Figure 1). These results are in agreement with those of other investigators who have shown that the potentiation of bone marrow toxicity is minimal when BSO is combined with adjuvant chemotherapy (Ono *et al.*, 1986; Ozols *et al.*, 1987; Tsutsui *et al.*, 1986; Russo *et al.*, 1986; Mitchell *et al.*, 1989). In our laboratories we have further observed that a single 2.5 mmol kg$^{-1}$ dose of BSO had little effect on either the acute animal lethality of doxorubicin (Lee *et al.*, 1987) or the lung toxicity of cyclophosphamide (Allalunis-Turner *et al.*, unpublished results). Given the observed increase in antitumour effect in the absence of enhanced bone marrow toxicity, it could be argued that our single dose BSO-MEL combination had resulted in an improved therapeutic index.

To determine whether a therapeutic gain could be achieved when multiple BSO exposures were combined with MEL, tumour response and bone marrow toxicity under these treatment conditions were assessed. Administering BSO four times prior to MEL, either at 16 or 6 h intervals, increased MEL antitumour efficacy ~1.3- and ~1.7-fold respectively (Figure 7b and 6c). This compares to an ER of ~1.4 observed when BSO was given as a single exposure 16 h prior to MEL (Figure 1). However, unlike the single dose of BSO pretreatment results, when administered as multiple exposures, not only did BSO appear to yield some stem cell toxicity on its own, but in addition led to enhancement of the marrow toxicity due to MEL (Figure 8b and 8c *vs* 8a). Thus administering BSO doses repeatedly at 16 h time intervals, to deplete tumour GSH prior to MEL treatment, led to little or no therapeutic gain. The 6 h pretreatment schedule did confer a large enhancement in the antitumour activity of MEL. However, due to the associated increase in bone marrow toxicity, the resultant therapeutic benefit was not larger than that seen with the single dose BSO-MEL combination.

The failure of the multi-dose BSO pretreatments to yield a significant therapeutic benefit most likely was related directly to the unfavourable bone marrow and tumour tissue GSH depletion and recovery kinetics resulting from these treatments strategies (see Discussion above). In contrast, when KHT sarcoma-bearing mice were kept on drinking water containing 30 mM BSO, the GSH levels in the tumours fell to 10–15% of control (Figure 5c) whereas those in the bone marrow were reduced by only ~10% (Figure 6c). When these animals were treated with MEL 4 days after starting the BSO treatment, the antitumour efficacy of this chemotherapeutic agent was found to be enhanced (~1.7-fold; Figure 7d) but the bone marrow toxicity was not increased (Figure 8d). Consequently, the drinking water treatment regimen led to the largest therapeutic gain in the present series of preclinical investigations using thiol depletion in conjunction with alkylating chemotherapy. This finding is comparable to enhancements seen in preclinical tumour models utilising a similar GSH depletion protocol in combination with hypoxic cell sensitisers and fractionated radiation treatments (Kramer *et al.*, 1989). These results suggest that chronic depletion of tumour GSH by prolonged BSO pretreatment ought to be considered in clinical trials of GSH modulation.

In conclusion, the evaluation of combinations of BSO with chemotherapy is currently a subject of extensive research. The present study has shown that it is possible in a model system to achieve a therapeutic benefit when BSO is combined with MEL if dose-timing and dose-sequencing are carefully evaluated. The BSO-MEL combination may be of particular interest since MEL remains one of the most effective single agents in the treatment of ovarian cancer. Our findings further suggest that, in clinical investigations aimed at modifying chemotherapeutic agent efficacy through thiol manipulations, there is a need to acquire detailed knowledge of the GSH depletion and recovery kinetics in critical normal tissues as well as tumours. No single normal tissue is likely to be representative of all others. Hence the best strategy may be to monitor the GSH content of a known dose-limiting tissue. Measurements of tumour GSH may be particularly critical and problematic given the difficulties associated with determining tumour GSH from biopsy specimens (Allalunis-Turner *et al.*, 1988; Mitchell *et al.*, 1989). Specifically, the regional variations in GSH measurements described for multiple biopsy samples taken from the same tumour (Allalunis-Turner *et al.*, 1988) need to be considered in the interpretation of clinical studies utilising thiol modulating agents. Despite these reservations, the experimental preclinical models strongly suggest that manipulation of tumour GSH may benefit selected patients.

This work was supported by NIH grant CA-11051. The authors thank A. Beikirch, D. Boyce and M. Chapman for expert technical assistance.

### References

ADAMS, D.J., CARMICHAEL, J. & WOLF, C.R. (1985). Altered tissue glutathione and glutathione transferase levels in response to cytoxins: studies with mouse bone marrow. *Cancer Res.*, **45**, 1669–1673.

ALLALUNIS-TURNER, M.J., LEE, F.Y.F. & SIEMANN, D.W. (1988). Comparison of glutathione levels in rodent and human tumor cells grown *in vitro* and *in vivo*. *Cancer Res.*, **48**, 3657–3660.

ARRICK, B.A. & NATHAN, C.F. (1984). Glutathione metabolism as a determinant of therapeutic efficacy: A Review. *Cancer Res.*, **44**, 4224–4232.

BIAGLOW, J.E., VARNES, M.E., CLARK, E.P. & EPP, E.P. (1983). The role of thiols in cellular response to radiation and drugs. *Radiat. Res.*, **95**, 437–455.

BRADLEY, T.R. & METCALF, D. (1966). The growth of mouse bone marrow cells *in vitro*. *Aust. J. Exp. Biol. Med. Sci.*, **44**, 287–299.

COLVIN, M., RUSSO, J.E., HILTON, J., DULIK, D.M. & FENSELAU, C. (1988). Enzymatic mechanisms of resistance to alkylating agents in tumor cells and normal tissue. *Adv. Enzyme Regulation*, **27**, 211–221.

CROOK, T.R., SOUHAMI, R.L., WHYMEN, G.D. & MCLEAN, A.E.M. (1986). Glutathione depletion as a determinant of sensitivity of human leukemia cells to cyclophosphamide. *Cancer Res.*, **46**, 5035–5038.

FARMER, P.B. (1987). Metabolism and reactions of alkyating agents. *Pharmac. Ther.*, **35**, 301–358.

HAMILTON, T.C., WINKER, M.A., LOUIE, K.G., BATIST, G., BEHRENS, B.C., TSURUO, T., GROTZINGER, K.R., MCKOY, N.M., YOUNG, R.C. & OZOLS, R.F. (1985). Augmentation of adriamycin, melphalan, and cisplatin cytotoxicity in drug-resistant and sensitive human ovarian carcinoma cell lines by buthionine sulfoximine mediated glutathione depletion. *Biochem. Pharmacol.*, **34**, 2583–2586.

HOSKING, L.K., WHELAN, R.D, SHELLARD, S.A., BEDFORD, P. & HILL, B.T. (1990). An evalution of the role of glutathione and its associated enzymes in the expression of differential sensitivities to antitumor agents shown by a range of human tumour cell lines. *Biochem. Pharm.*, **40**, 1833–1842.

KRAMER, R.A., GREENE, K., AHMAD, S. & VISTICA, D.T. (1987). Chemosensitization of L-phenylalanine mustard by the thiol-modulating agent buthionine sulfoximine. *Cancer Res.*, **47**, 1593–1597.

KRAMER, R.A., SOBEL, M. & MONTOYA, V.P. (1989). The effect of glutathione (GSH) depletion *in vivo* by buthionine sulfoximine (BSO) on the radiosensitization of SR 2508. *Int. J. Radiat. Oncol. Biol. Phys.*, **16**, 1325–1329.

LEE, F.Y.F., ALLALUNIS-TURNER, M.J. & SIEMANN, D.W. (1987). Depletion of tumour *vs* normal tissue glutathione by buthionine sulfoximine. *Br. J. Cancer*, **56**, 33–38.

LEE, F.Y.F., ALLALUNIS-TURNER, M.J., KENG, P.C. & SIEMANN, D.W. (1988). Glutathione contents in human and rodent tumor cells in various phases of the cell cycle. *Cancer Res.*, **48**, 3661–3665.

LEE, F.Y.F., SIEMANN, D.W. & SUTHERLAND, R.M. (1989). Changes in cellular glutathione content during adriamycin treatment in human ovarian cancer, a possible indicator of chemosensitivity. *Br. J. Cancer*, **60**, 291–298.

LEE, F.Y.F., FLANNERY, D.J. & SIEMANN, D.W. (1991). Prediction of tumour sensitivity to 4-hydroperoxycyclophosphamide by a glutathione-targeted assay. *Br. J. Cancer*, **63**, 217–222.

MEISTER, A. (1983). Selective modification of glutathione metabolism. *Science*, **220**, 472–477.

MINCHINTON, A.I., ROJAS, A., SMITH, K.A. & SORANSON, J.A. (1984). Glutathione depletion in tissues after administration of buthionine sulfoximine. *Int. J. Radiat. Oncol. Biol. Phys.*, **10**, 1261–1264.

MITCHELL, J.B., COOK, J.A., DEGRAFF, W., GLATSTEIN, E. & RUSSO, A. (1989). Glutathione modulation in cancer treatment: will it work? *Int. J. Radiat. Oncol. Biol. Phys.*, **16**, 1289–1295.

MORSTYN, G., RUSSO, A., CARNEY, D., KARAWA, E., WILSON, S.H. & MITCHELL, J.B. (1984). Heterogeneity in the radiation survival curves and the biochemical properties of human lung cancer lines. *JNCI*, **73**, 801–807.

ONO, K., KOMURO, C., TSUTSUI, K., SHIBAMOTO, Y., NISHIDAI, T., TAKAHASHI, M., ABE, M. & SHRIEVE, D.C. (1986). Combined effect of buthionine sulfoximine and cyclophosphamide upon murine tumours and bone marrow. *Br. J. Cancer*, **54**, 749–754.

OZOLS, R.F. (1985). Pharmacological reverse of drug resistance in ovarian cancer. *Seminars in Oncol.*, **7**, 7–11.

OZOLS, R.F., LOUIE, K.C., PLOWMAN, J., BEHRENS, B.C., FINE, R.L., DYKES, D. & HAMILTON, T.C. (1987). Enhanced melphalan cytotoxicity in human ovarian cancer *in vitro* and in tumor-bearing nude mice by buthionine sulfoximine depletion of glutathione. *Biochem. Pharmacol.*, **36**, 147–153.

RICHARDSON, M.E. & SIEMANN, D.W. (1992). Thiol manipulation as a means of overcoming drug resistance in a novel cyclophosphamide-induced resistant cell line. *Int. J. Radiat. Oncol. Biol. Phys.*, **22**, 781–784.

RUSSO, A., TOCHNER, Z., PHILLIPS, T., CARMICHAEL, J., DEGRAFF, W., FRIEDMAN, N., FISHER, J. & MITCHELL, J.B. (1986). *In vivo* modulation of glutathione by buthionine sulfoximine: effect on marrow response to melphalan. *Int. J. Radiat. Oncol. Biol. Phys.*, **12**, 1187–1189.

SUZUKAKE, K., PETRO, B.J. & VISTICA, D.T. (1982). Reduction in glutathione content of L-Pam resistant L1210 cells confers drug sensitivity. *Biochem. Pharmacol.*, **31**, 121–124.

THOMSON, J.E. & RAUTH, A.M. (1974). An *in vitro* assay to measure the viability of KHT tumor cells not previously exposed to culture conditions. *Radiat. Res.*, **58**, 262–276.

TSUTSUI, K., KOMURO, C., ONO, K., NISHIDAI, T., SHIBAMOTO, Y., TAKAHASHI, M. & ABE, M. (1986). Chemosensitization of buthionine sulfoximine *in vivo*. *Int. J. Radiat. Oncol. Biol. Phys.*, **12**, 1183–1186.